# Mortality in patients with major depressive disorder: A nationwide population-based cohort study with 11-year follow-up

**DOI:** 10.1192/j.eurpsy.2024.1771

**Published:** 2024-09-30

**Authors:** Istvan Bitter, Gyorgy Szekeres, Qian Cai, Laszlo Feher, Judit Gimesi-Orszagh, Peter Kunovszki, Antoine C. El Khoury, Peter Dome, Zoltan Rihmer

**Affiliations:** 1 Department of Psychiatry and Psychotherapy, Semmelweis University, Budapest, Hungary; 2 Department of Psychiatry and Psychotherapy, Saint Rókus Hospital, Semmelweis University, Budapest, Hungary; 3 Janssen Global Services, LLC, Titusville, NJ, USA; 4 Janssen-Cilag Kft., Budapest, Hungary; 5 Janssen Global Services, Budapest, Hungary; 6 Nyiro Gyula National Institute for Psychiatry and Addictology, Budapest, Hungary

**Keywords:** bipolar conversion, comorbidity, major depressive disorder, mortality, schizophrenia

## Abstract

**Background:**

Major depressive disorder (MDD) is a leading cause of disability and premature mortality. This study compared the overall survival (OS) between patients with MDD and non-MDD controls stratified by gender, age, and comorbidities.

**Methods:**

This nationwide population-based cohort study utilized longitudinal patient data (01/01/2010 – 12/31/2020) from the Hungarian National Health Insurance Fund database, which contains healthcare service data for the Hungarian population. Patients with MDD were selected and matched 1:1 to those without MDD using exact matching. The rates of conversion from MDD to bipolar disorder (BD) or schizophrenia were also investigated.

**Results:**

Overall, 471,773 patients were included in each of the matched MDD and non-MDD groups. Patients with MDD had significantly worse OS than non-MDD controls (hazard ratio [HR] = 1.50; 95% CI: 1.48−1.51; males HR = 1.69, 95% CI: 1.66–1.72; females HR = 1.40, 95% CI: 1.38–1.42). The estimated life expectancy of patients with MDD was 7.8 and 6.0 years less than that of controls aged 20 and 45 years, respectively. Adjusted analyses based on the presence of baseline comorbidities also showed that patients with MDD had worse survival than non-MDD controls (adjusted HR = 1.29, 95% CI: 1.28–1.31). After 11 years of follow-up, the cumulative conversions from MDD to BD and schizophrenia were 6.8 and 3.4%, respectively. Converted patients had significantly worse OS than non-converted patients.

**Conclusions:**

Compared with the non-MDD controls, a higher mortality rate in patients with MDD, especially in those with comorbidities and/or who have converted to BD or schizophrenia, suggests that early detection and personalized treatment of MDD may reduce the mortality in patients diagnosed with MDD.

## Introduction

Major depressive disorder (MDD), with a lifetime risk of 15 –18%, is a leading cause of disability and premature mortality affecting around 300 million (4.4%) people worldwide, and projected to be ranked as the first cause of the global burden of disease in 2030 [1–5]. In 2021, the 1-year prevalence of depression was around 7% in Hungary [6, 7]. MDD has a significant negative impact on the quality of life, daily activities, cognitive functions, employment status, and work productivity of patients due to the complexity of MDD and MDD-associated comorbidities [8].

Despite progress in the treatment of depression, high mortality rates associated with depressive disorders have been observed [9–12]. Several secondary database studies from various countries found that patients with depressive disorders were at a higher risk of overall mortality than the general population [9–11, 13–15]. In addition to suicidality, MDD is associated with several comorbidities, including cardiovascular, metabolic, and endocrine diseases, and addictive disorders (e.g., smoking and use of other substances), and poor medical treatment compliance [16]. Natural (e.g., somatic comorbidities) and external causes (e.g., suicide, accidents) are responsible for excessive mortality and shortened life expectancy in patients with MDD [10, 12, 14, 15, 17]. Patients with comorbidities (e.g., cancer and cardiovascular diseases) and depression were at a higher risk of mortality than patients with the same comorbidities without depression [18–20]. In a meta-analysis, Cuijpers et al. found higher mortality in depressed individuals than in nondepressed individuals and emphasized that the relative risk of mortality was the same in higher- and lower-prevalence populations. Their study highlighted that not disease-specific mechanisms (depression-related increased platelet aggregation, which is considered a risk factor for heart disease) but general mechanisms (e.g., unhealthy lifestyle, which is more common in individuals with MDD and associated with general adverse health effects and can lead to various somatic illnesses) were the most likely underlying causes of excess mortality in depression [21].

Retrospective and prospective studies have confirmed that major depressive episode is the first manifestation of bipolar disorder (BD) in 50–80% of individuals with the final diagnosis of BD [22, 23]. In these patients, the definitive diagnosis of BD can be established when the manic/hypomanic episode(s) occur later in life [24]. The 15-year cumulative incidence rates of conversion from MDD to BD, schizophrenia, and schizoaffective disorder were reported to be 7.4, 2.5, and 1.3%, respectively [25]. Additionally, the conversion rates to BD and schizophrenia were typically higher in the early course of the illness [25, 26]. Considerable research has been devoted to evaluating the survival and mortality of patients with mental disorders, including MDD, BD, and schizophrenia, including studies using national healthcare databases [9–11, 13–15, 17, 27, 28].

The primary objective of this study was to estimate the overall survival (OS) of patients with a primary diagnosis of MDD compared to a matched control group (non-MDD) selected from the Hungarian nationwide insurance database. The secondary objectives included: (1) to evaluate the OS in MDD versus non-MDD stratified by baseline comorbid conditions of interest and (2) to estimate the OS in patients with MDD who converted to BD or schizophrenia.

## Methods

### Data sources

This was a nationwide, register-based prospective matched cohort study using data between January 1, 2010, and December 31, 2020. The study utilized longitudinal patient data from the Hungarian National Health Insurance Fund (NHIF) database, which is a nationwide, government-owned health insurance database and covers detailed healthcare service data for almost the entire Hungarian population (approximately 10 million people). The database includes demographic data (e.g., date of birth, gender, and geographical region) of all patients, and all recorded healthcare events are linked to individual patients by a unique patient identifier (social security number). The NHIF database also encompasses financial claims information for all inpatient hospital stays and outpatient visits and for all pharmacy-dispensed prescriptions. The International Classification of Diseases, Tenth revision (ICD-10) was used to define the diagnoses of interest (Supplementary Table S1) [29] and the Anatomical Therapeutic Chemical classification system was used to code dispensed prescriptions [30]. Furthermore, patient death information was collected rigorously, and the death dates of all deceased patients were recorded with no missing data in the NHIF database. This study was approved by the Medical Research Council – Research and Ethics Committee (TUKEB), Hungary (Approval number: IV/4021-3/2022/EKU dated May 31, 2022).

### Study population

**Figure 1.**
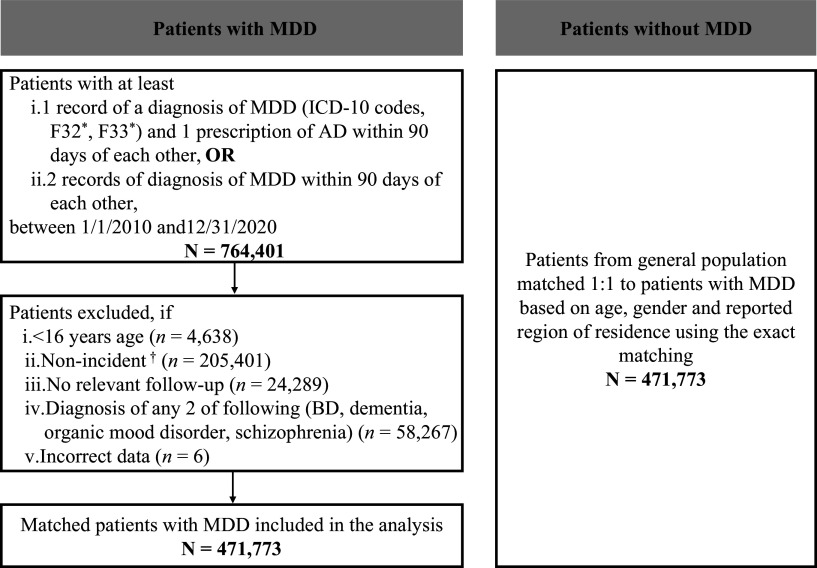
Flow diagram of patient selection. *ICD-10 codes. The following comorbidities were only assessed in inpatient care (as primary reason for hospitalization and secondary diagnoses) and a single report of the code is sufficient. ^†^Patients with a diagnosis of MDD or a prescription of AD within 365 days prior to the MDD index date. Abbreviations: AD, antidepressant drug; BD, bipolar disorder; ICD-10, International Classification of Diseases Version 10; MDD, major depressive disorder.

In the period between January 1, 2010 and December 31, 2020, patients with MDD were selected if they had at least (i) one record of a diagnosis of MDD (ICD-10 F32* [MDD, single episode], F33* [MDD, recurrent episode]) and 1 prescription of antidepressant drug (AD) within 90 days of each other, or (ii) two records of the diagnosis of MDD within 90 days of each other (Figure 1). The index date was defined as the date of the first observed MDD diagnosis or AD prescription, whichever occurred earlier. Patients with an ongoing depressive episode at the time of the start of data availability were excluded as the requirement was to have a ≥1-year MDD-free period (i.e., no MDD and no AD prescriptions) prior to the index date; thus, all analyzed patients were followed up from the start of a new depressive episode. Patients were also required to have ≥1-year follow-up before the end of data availability (i.e., the index date was required to be on or before December 31, 2019). Patients aged <16 years as of the index date were excluded. Individuals without a diagnosis of MDD (i.e., non-MDD) were also selected from the NHIF database and matched 1:1 with patients with MDD based on age, gender, and reported region of residence using exact matching. This matching was used for all comparisons between patients with MDD and non-MDD controls. The index date of the non-MDD controls was defined based on the index date of the patients with MDD to whom they were matched.

As certain comorbidities have a potential effect on MDD mortality [2, 31], patients with MDD and non-MDD controls were, respectively, stratified into two groups based on the presence or absence of comorbidities within 1 year prior to the index date.

Furthermore, among patients with MDD, those with diagnostic conversions from MDD to BD or MDD to schizophrenia were matched to those without experiencing conversion using a 1:1 propensity score matching algorithm. Propensity scores were generated using multivariate logistic regression with covariates of age, gender, and presence or absence of comorbidities within 1 year prior to the index date [32].

### Study assessments

The study compared the OS between patients with MDD and non-MDD controls. The OS was defined as the time from the index date until the death of the patient due to any cause. The OS was also evaluated by gender and age groups (16–20, 21–40, 41–60, 61–80, >80 years) for the comparison groups, and the corresponding life tables were constructed. The life expectancy was calculated using the life tables, which were constructed using 5-year intervals of age assuming that survival within the age categories was the same regardless of age [33].

To reduce potential bias, stratified analyses were performed to compare OS between patients with MDD and non-MDD controls, stratified by the presence or absence of baseline comorbidities of interest. To better understand the OS of patients with MDD who converted to BD or schizophrenia, the percentage of patients with converted disorders over time was estimated. The OS was then compared between patients with MDD with BD or schizophrenia conversion and matched patients who did not experience such conversion.

### Statistical analysis

Continuous variables were summarized by means, standard deviations, medians, and interquartile ranges. Categorical variables were presented as frequencies and percentages. The *t* tests and Chi-square tests were used for continuous variables and categorical variables, respectively, to compare differences in the study outcomes between the comparison groups. The Kaplan–Meier estimation was used to estimate the survival, and the log-rank test was used to evaluate the differences in survival distributions of the comparison groups. The Cox proportional hazard models were used to estimate the hazard ratio (HR) of mortality between the comparison groups. In addition, among patients with MDD, the cumulative incidence of subsequent diagnosis of BD or schizophrenia was estimated from the index date. Data analyses were performed using R statistical software (version 4.2.2) [34].

## Results

A total of 471,800 patients with MDD who met the inclusion and exclusion criteria were selected (Figure 1). After the exact matching, 471,773 patients with a mean (standard deviation [SD]) age of 54.8 (17.4) years and 67.2% females were included in each of the matched MDD and non-MDD groups. Compared with the matched non-MDD controls, the MDD group had a significantly higher percentage of patients with various comorbidities during the 12-month pre-index period (Table 1).

**Table 1.** Baseline characteristics of patients with MDD and matched non-MDD controls.

	MDD (N = 471,773)	Matched non-MDD (N = 471,773)	p-Value
**Age, years**			
Mean (SD)	54.8 (17.4)	54.8 (17.4)	1.000
Median	55.3	55.3	
Range (min, max)	(16.0, 107.4)	(16.0, 107.4)	
Female, %	67.2	67.2	1.000
**Comorbidities, %**			
Malignant neoplasms	6.8	4.0	<0.001
Cerebrovascular and cardiovascular diseases, atherosclerosis, and pulmonary embolism	31.7	16.7	<0.001
Hypertensive diseases	52.7	37.7	<0.001
Acute lower respiratory infections	6.8	3.2	<0.001
Bronchitis, emphysema, asthma	10.4	5.2	<0.001
Diabetes mellitus	12.1	8.2	<0.001
Liver disease, including viral hepatitis	1.8	0.7	<0.001
Epilepsy	2.0	0.8	<0.001
Substance use disorders	2.8	0.3	<0.001
Anxiety and stress disorders	30.7	4.0	<0.001
Personality disorders	0.8	0.1	<0.001
Serious infections	1.6	0.5	<0.001
Accidents and other external causes of morbidity	17.6	9.9	<0.001
Suicide attempt/self–harm	1.2	0.1	<0.001

Abbreviations: MDD, major depressive disorder; SD, standard deviation.

### OS and expected life expectancy between MDD and non-MDD

**Figure 2.**
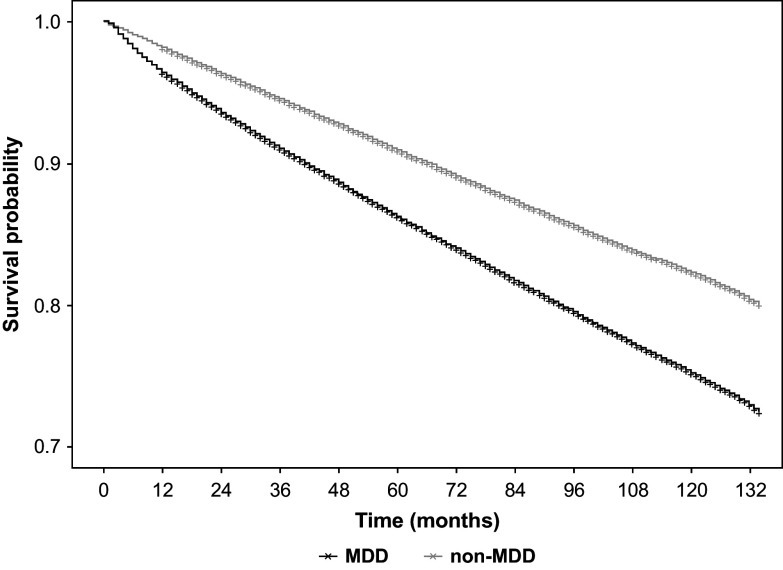
Overall survival between patients with MDD and matched non-MDD controls. Abbreviations: MDD, major depressive disorder.

During the 11-year observation period (January 1, 2010–December 31, 2020), the OS of patients with MDD was significantly worse than that of the matched non-MDD controls (unadjusted HR = 1.50; 95% confidence interval [CI]: 1.48–1.51; *p* < 0.001, Figure 2). When stratified by gender, male (HR = 1.69; 95% CI: 1.66–1.72) and female (HR = 1.40; 95% CI: 1.38–1.42) patients with MDD had an increased risk of death compared with non-MDD controls. After adjusting for baseline comorbidities, patients with MDD still had a worse OS compared with matched non-MDD controls (adjusted HR = 1.29; 95% CI: 1.28–1.31; *p* < 0.001).

The estimated 5-year mortality rates increased with age; within each age group, males had a higher estimated 5-year mortality rate than females in both the MDD and non-MDD groups. At age 20, the estimated life expectancy difference between the MDD and non-MDD groups (in favor of non-MDD controls) was 7.8 years (10.2 years in males and 5.9 years in females), whereas at age 45, it was 6.0 years (7.8 years in males and 4.6 years in females).

### OS between MDD and non-MDD stratified by comorbidities of interest

**Figure 3.**
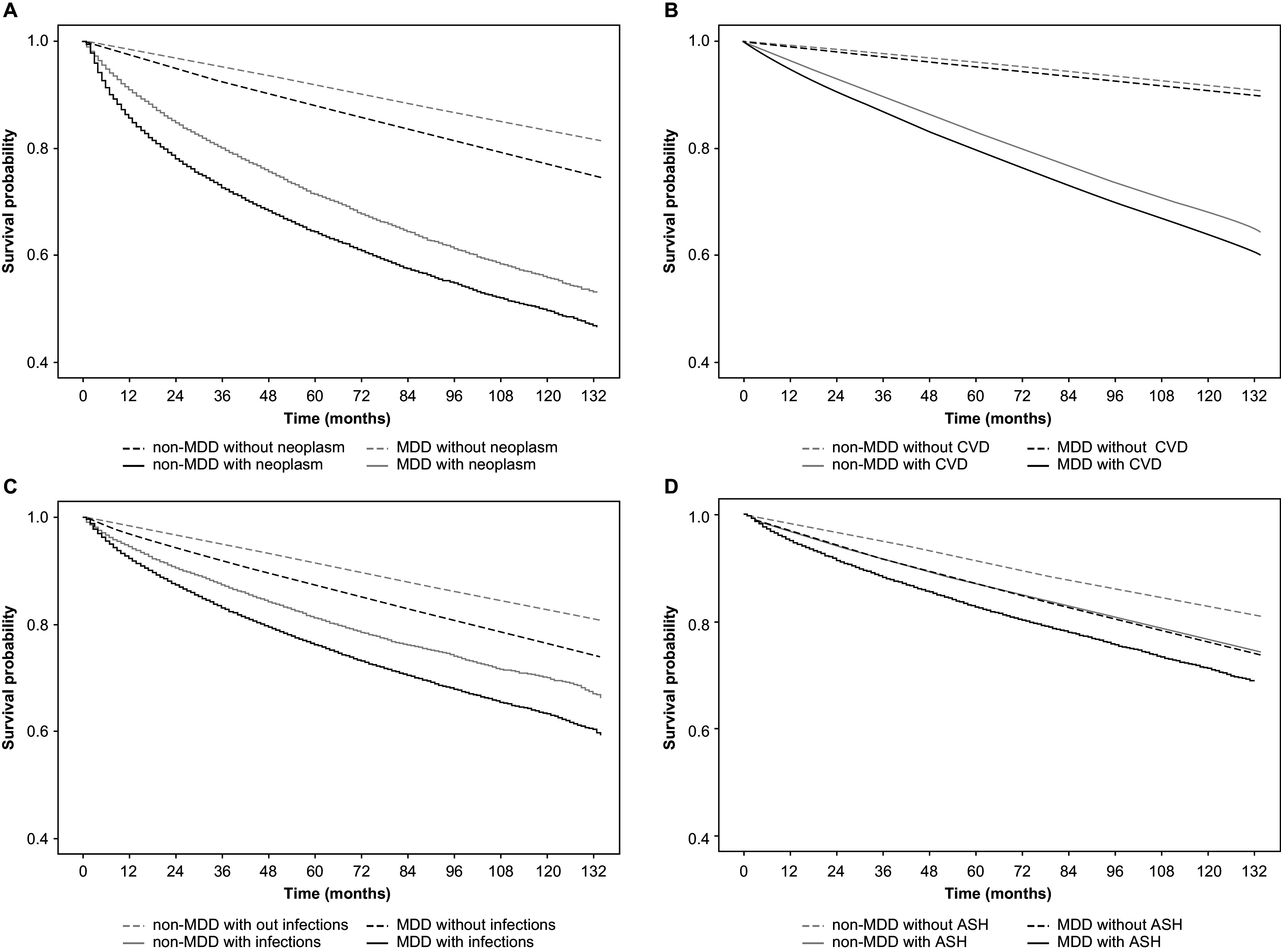
Comparison of overall survival between patients with MDD and matched non-MDD controls stratified by the presence/absence of malignant neoplasms (A), cardiovascular and cerebrovascular disorder (CVD) (B), infections (C), and accidents and self-harm (ASH) (D). Abbreviations: MDD, major depressive disorder.

Stratified analysis by baseline comorbidities of interest yielded similar findings. During the 11-year observation period, the OS of patients with MDD was significantly worse than that of the matched non-MDD controls in all strata, including malignant neoplasms, cardiovascular and cerebrovascular diseases, infections, suicide attempts, and self-harm (Figure 3).

### OS of patients with MDD with conversion and non-conversion

**Figure 4.**
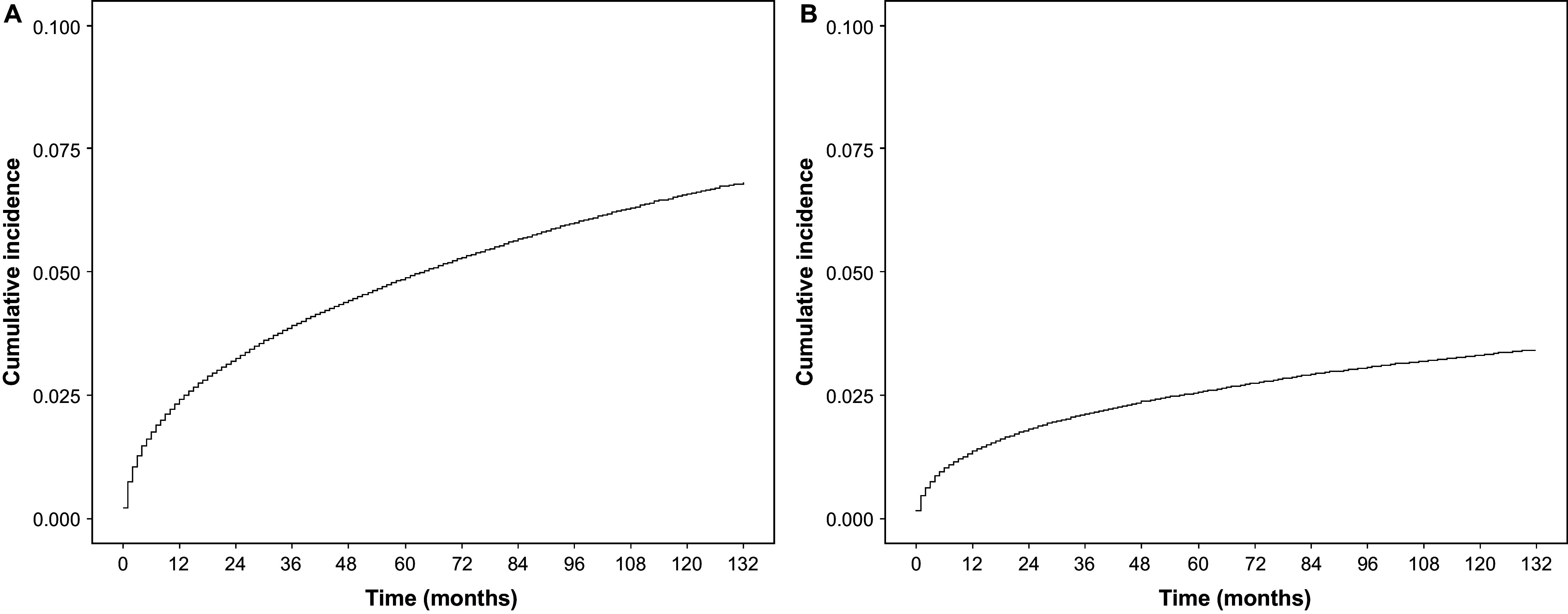
Cumulative incidence of conversion of patients with MDD to bipolar disorder (A), and schizophrenia (B). Abbreviations: MDD, major depressive disorder.

Among patients with an initial diagnosis of MDD, 2.4% had a diagnosis of BD by the end of the first year, increasing to 3.3% by the end of the second year, 4.9% by the end of the fifth year, and 6.8% by the end of the eleventh year. A similar trend was observed for conversion to schizophrenia from an initial diagnosis of MDD: 1.4% had a diagnosis of schizophrenia by the end of the first year, 1.8% by the end of the second year, 2.6% by the end of the fifth year, and 3.4% by the end of the eleventh year (Figure 4). It was observed from the slope of the curve that the rate of conversion to either BD or schizophrenia was the highest immediately after the diagnosis of MDD and decreased over time.

**Figure 5.**
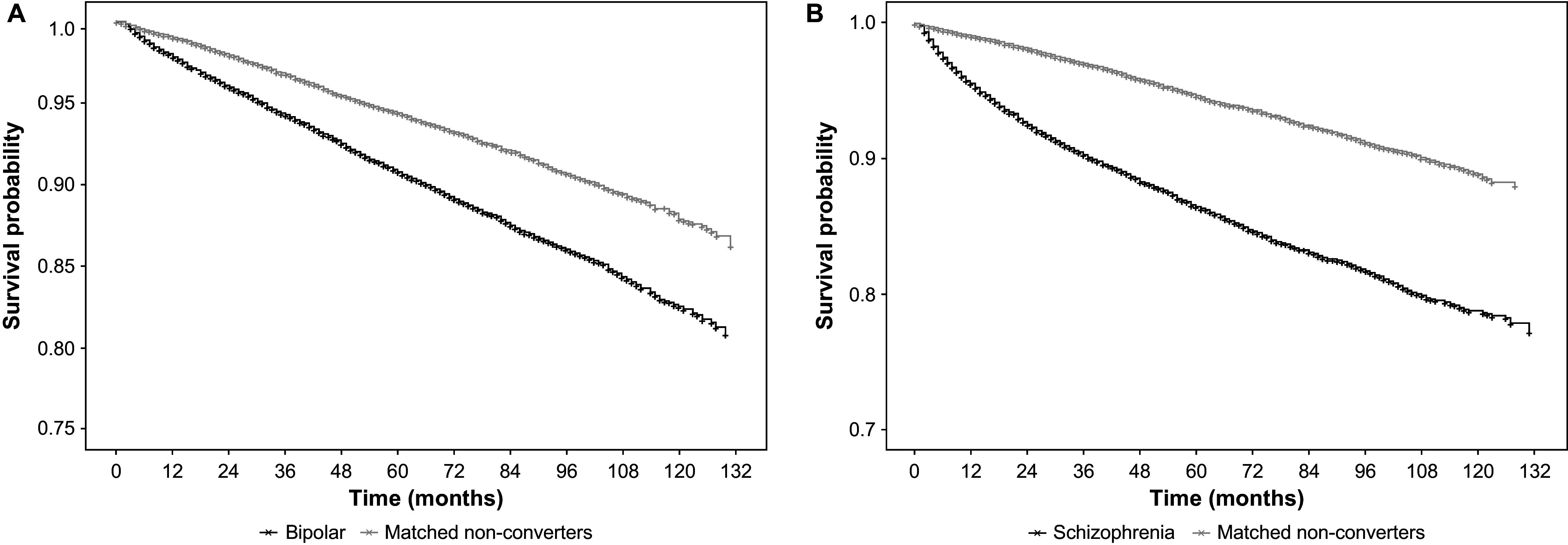
Comparison of overall survival between patients with MDD converting to bipolar disorder (A), or schizophrenia (B) and the matched non-converters. Abbreviations: MDD, major depressive disorder.

A total of 23,899 and 12,257 patients with MDD who converted to BD and schizophrenia, respectively, were further matched to patients without experiencing the conversion using 1:1 propensity score matching. Patients with MDD who converted to BD (HR = 1.61; 95% CI: 1.51–1.72; *p* < 0.001) and schizophrenia (HR = 2.48; 95% CI: 2.31–2.67; *p* < 0.001) had a worse OS than those without conversion (Figure 5).

## Discussion

This nationwide real-world study using a national healthcare database analyzed the OS and the estimated life expectancy at 20 and 45 years of age in patients with MDD versus the matched non-MDD controls. Additionally, the study evaluated the OS in patients with MDD versus non-MDD stratified by baseline comorbidities of interest, and in patients with MDD who converted to BD or schizophrenia. In the 11-year observation period, patients with MDD had a significantly higher mortality rate than the non-MDD controls in both genders. With increasing age, the 5-year mortality rates were increased in both the MDD and non-MDD controls, but the increase was more pronounced in patients with MDD. When stratified by selected baseline comorbidities, patients with MDD had a lower rate of OS compared with non-MDD controls. Furthermore, a decreasing rate of conversion from MDD to BD or schizophrenia was observed over time, and patients with MDD who converted to BD or schizophrenia had a higher risk of mortality than those with MDD patients without the conversion.

MDD is one of the frequent psychiatric disorders, and research has consistently shown that patients with MDD are at a higher risk of all-cause mortality than the general population [9, 11, 35, 36]. In the current study, even after adjusting for baseline comorbidities, patients with MDD had a 29% higher risk of mortality than the age/gender/region of the residence-matched non-MDD population during the 11-year observation period. Similar findings on mortality were reported from the Helsinki birth cohort study (N = 1,995) [9]. A retrospective Spanish study (N = 4,583) revealed that patients with depression had a 50% increased risk of mortality versus the non-depressive population [13]. A Danish population-based cohort study (1995–2013) found that the overall mortality is about two times higher in patients with MDD compared with the general Danish population, which translated into a reduced life expectancy of 10–14 years in these patients [14]. It is worth noting that relatively few studies have been conducted in the East-Central European countries to assess excess mortality among patients with mood disorders (including MDD) [37, 38]. A nationwide, register-based cohort study in Poland showed a slightly elevated standardized annual mortality ratio (SMR = 1.08; 95% CI: 1.05–1.10) for subjects with any disorder in the ICD-10 diagnostic group “mood (affective) disorders” (F30–F39) compared to the general population [37]. Results from a study in the Czech Republic showed that people who were discharged from mental health institutions with any of the ICD-10 codes corresponding to mood (affective) disorders (F30–F39) had higher mortality (SMR = 1.6; 95% CI: 1.5–1.7) than the general population [38]. However, the results of these two studies cannot be directly compared with our results because (1) in both studies, SMR was used to express the extent of excess mortality in subjects with mental disorders (in contrast to our study, where HR was used for the same purpose); (2) the Czech study included only individuals who were treated as inpatients in a psychiatric institution and then discharged; (3) both studies included patients who may have suffered from any mood disorder, whereas we included only those with a baseline diagnosis of MDD [37, 38]. The survival results of the present study are consistent with the findings from the national representative populations across different regions of the world [21], providing further evidence that patients with MDD are at an increased risk of death.

In the stratified analysis, although both males and females with MDD had a higher mortality rate compared with the non-MDD population, males had a slightly higher risk than females. These findings are in line with the published studies highlighting the increased mortality in depressed males [11, 14, 16, 39]. In this study, in addition to nonfatal suicidal behavior, accidents, and self-harm, somatic comorbidities, such as cardio-cerebrovascular diseases, neoplasms, and infections, were associated with higher mortality in patients with MDD compared with non-MDD controls. Comorbid depression in somatic disease is associated with poor quality of life, worse course of the disease, higher functional impairment and disability, increased service utilization, higher medical costs, and increased patient mortality compared to the presence of either depression or the somatic disease alone [18–20, 25–27, 31, 35, 36, 40–46]. Depression has been recognized as a risk factor for the studied somatic diseases (especially for cardiovascular disorders and infections; with regard to malignancies, the results are inconclusive) and other somatic diseases, including diabetes, hypertension, and rheumatoid arthritis [2, 42, 43, 47–49]. On the other hand, several kinds of somatic disorders have been recognized as key risk factors for major depression [18, 42–44, 50]. Additionally, a recent Swedish population-based cohort study including >145,000 patients with unipolar MDD reported that patients with MDD with records of suicidal behavior had a higher risk of all-cause mortality (HR = 2.62, 95% CI: 2.15–3.20) compared with the matched non-suicidal MDD controls [51].

The increased risk of mortality in patients with depression might be due to a greater inclination toward adverse health behaviors, noncompliance with medical treatment leading to poor health outcomes, and disease progression [52–55]. Thus, appropriate medical treatment for depression could reduce morbidity and mortality in patients with depression and comorbidities [45, 46, 56, 57]. Additionally, previous studies demonstrated that appropriate patient education and training regarding the use of antidepressant medications could aid in reducing the mortality and improving the clinical condition and quality of life in MDD patients with comorbidities [45, 46, 56, 57].

The conversion rates from MDD to BD vary in different studies; however, the majority reported more frequent conversion at a young age and decreasing rates over time [22, 58]. In line with the results of the present study, in a Finish prospective nationwide register-based cohort (N = 43,495), of all first psychiatric hospitalizations due to unipolar depression, the 15-year cumulative conversion rate to BD was 7.4% [25]. A Swedish national longitudinal study showed that during a 13-year follow-up of 641,064 patients with unipolar major depression, the cumulative incidence of conversion to BD was 5.8% [59]. Similar to the review of Kessing et al. [26] and some more recent clinical studies [25], the present study also showed that the yearly incidence of bipolar conversion decreased over time: 2.4% in the first year, 0.9% in the second year, and <1% in each of the following 9 years. Our study also showed a decreasing trend in the yearly conversion rate from MDD to schizophrenia (1.4% in the first year, 0.4% in the second year, and <0.5% in each subsequent year till 11 years). Similar to the present study, Musliner et al. found a decreasing risk of conversion of MDD to schizophrenia over 10 years in a study of 71,932 Danish patients [60]. Other studies have also found that the rate of conversion from MDD to schizophrenia is highest in the first year after the diagnosis of unipolar depression and decreasing over time [25].

Although conversions have been well documented in the literature, there is a paucity of data on the mortality rate differences between the converted and non-converted patients from MDD to BD or schizophrenia. The present analyses showed an increased death risk of 61 and 148% in patients who converted from MDD to BD and schizophrenia, respectively, compared with non-converted patients. This is in agreement with the finding that the overall mortality rate of patients with BD is higher than those with unipolar depression, and with findings that schizophrenia is associated with higher all-cause mortality than MDD or BD [61, 62]. Therefore, it is important to screen patients with MDD for symptoms of BD and schizophrenia to identify these disorders early and provide appropriate treatment, which could potentially reduce the incidence of mortality [40, 63, 64]. Supplementary analysis showed that the OS of the non-converted patients with MDD were similar to that of the entire MDD patient population (i.e., non-converters and converters together), which was unsurprising as non-converters far outnumbered converters.

The present study has limitations similar to other studies based on administrative healthcare data. The data collected from the Hungarian NHIF database serves financial and reimbursement purposes; however, it is not used for clinical evaluation. Therefore, clinical data, such as laboratory values, disease severity indices, and patient-reported outcomes were not available. The cause of death was not available and/or could not be determined from this database. Psychotherapy provided in private outpatient practices was not recorded in the database; however, medication prescriptions from private care were available when filled at any pharmacy. Propensity score matching was used to balance the cohorts based on known factors and address biases resulting from observed covariates; however, it did not account for bias that might stem from unobserved covariates that could influence the outcomes. Finally, the exclusion criterion that patients had to have a period of ≥1 year free of MDD (i.e., no MDD and no AD prescription) prior to the index date may have resulted in selection bias, as the application of this criterion implied that we excluded a subset of patients with chronic, recurrent, or treatment-resistant depression (TRD). Subjects in different age cohorts would have different odds of becoming members of the TRD group. This was one of the reasons why the analysis of the data of the TRD subgroup was not included in this research. However, an unknown proportion of the 471,800 patients with MDD in the study developed a chronic course or TRD during the 11 years of follow-up, and their data were included in our analyses.

Despite the limitations, the notable strengths of the study are the large sample size and the use of a database with nationwide coverage; this encompasses all major segments of healthcare and provides longitudinal follow-up data. Furthermore, the ascertainment of death is of high quality in the NHIF database since information on death is important for the discontinuation of patient insurance.

In conclusion, this study evaluated the OS in patients with MDD compared with non-MDD controls, along with stratified analyses based on gender, age, and comorbidities of interest, as well as among patients with conversion to BD or schizophrenia using real-world patient data from a nationwide insurance database. Patients with MDD had a higher risk of death than non-MDD population. Male patients had slightly worse survival than female patients with MDD. Overall, patients with MDD with baseline comorbidities had an additional increase in the risk of mortality as compared to patients with MDD having no comorbidities and controls with given comorbidity but without MDD. In addition, patients with MDD who converted to BD or schizophrenia had worse survival than patients with MDD without the conversion; therefore, early detection may be requisite to reduce mortality in these two subpopulations. Along with the existing evidence on survival outcomes in MDD versus non-MDD, these findings could help public health endeavors to build a comprehensive approach for the overall mental and social improvements in patients diagnosed with MDD.

## Supporting information

Bitter et al. supplementary material Bitter et al. supplementary material

## Data Availability

The data sharing policy of Janssen Pharmaceutical Companies of Johnson & Johnson is available at https://www.janssen.com/clinical-trials/transparency. These data were used under license for the current study and are not publicly available. Other researchers should contact the Hungarian National Health Insurance Fund (NHIF).

## Supplementary material

The supplementary material for this article can be found at http://doi.org/10.1192/j.eurpsy.2024.1771.
