# Supplementary material for: Mortality in patients with major depressive disorder: A nationwide population-based cohort study with 11-year follow-up
Source: Eur Psychiatry. 2024 Sep 30;67(1):e63. doi: 10.1192/j.eurpsy.2024.1771 (PMC11536202; doi:10.1192/j.eurpsy.2024.1771)
Supplement: Bitter et al. supplementary material [file S0924933824017711sup001.docx]

**Supplementary Appendix**

**Table S1.** The list of ICD-10 codes used in the study

| **Disease** | **ICD-10 codes** |
| --- | --- |
| MDD, single | F32^*^ |
| MDD, recurrent | F33^*^ |
| Malignant neoplasms | C^*^, D00 – D09, D37 – D48 |
| Cardiovascular diseases, atherosclerosis and pulmonary embolism | I20 – I26, I60 – I69, I70, G45 |
| Hypertensive diseases | I10 – I15 |
| Acute lower respiratory infections | J09 – J22 |
| Bronchitis, emphysema, asthma | J40 – J46 |
| Diabetes mellitus | E10 – E14 |
| Liver disease, including viral hepatitis | B15 – B19, K70 – K77 |
| Epilepsy | G40, G41 |
| Substance use disorders | F10 – F19 |
| Anxiety and stressor-related disorders | F40 – F43 |
| Personality disorders | F60, F61 |
| Serious infections^*^ | A00 – A99, B00 – B09, B20 – B99 |
| Accidents and other external causes of morbidity^†^ | S00 – S99, T00 – T98, V01 – V99, W00 – W99, X00 – X59, X85 – X99, Y01 – Y09 |
| Suicide attempt / self-harm^†^ | X60 – X84, Y10 – Y34 |

^*^The following comorbidities were only assessed in inpatient care (as primary reason for hospitalization and secondary diagnoses) and a single report of the code is sufficient.

^†^The following comorbidities were only assessed in inpatient care (as primary reason for hospitalization and secondary diagnoses) and in outpatient care and a single report of the code is sufficient.

Abbreviations: ICD-10, International Classification of Diseases Version 10; MDD, major depressive disorder.

When comorbidities were assessed, the patient needed to have at least 2 records of the codes in the inpatient, outpatient, or prescription dataset for the patient to be considered as having said comorbidity.
